# The prevalence and etiology of anemia and the association between anemia and all-cause mortality: a cohort study over a 9-year period

**DOI:** 10.1186/s12877-025-06353-2

**Published:** 2025-09-26

**Authors:** Yazhi Yang, Hurong Lai, Caifeng Liao, Huijun Chen, Xiaoyan Jiang, Chuyang Lin, Ling He, Huaijun Tu, Jian LI

**Affiliations:** 1 The Second Affiliated Hospital, Jiangxi Medical College, Nanchang University, Nanchang, Jiangxi China; 2 Department of Clinical Research Center, The Second Affiliated Hospital of Nanchang University, Nanchang, Jiangxi China; 3 Department of Geriatrics, The Second Affiliated Hospital of Nanchang University, Nanchang, Jiangxi China

**Keywords:** Anemia, Elderly, Quality of life, Mortality, Risk factors, Cohort

## Abstract

**Research Background:**

As the global population ages rapidly, anemia prevalence rises among older adults, yet its impact on quality of life and mortality in this group remains poorly understood. This study aimed to identify risk factors for anemia and examine its associations with quality of life and 9-year all-cause mortality among Chinese adults aged ≥60.

**Methods:**

We evaluated the determinants and consequences of anemia in 5,050 participants aged ≥ 60 from the China Health and Retirement Longitudinal Study (CHARLS), a prospective cohort of community-dwelling elderly individuals ≥ 45 at baseline and followed for up to 9 years. Anemia was defined according to the World Health Organization criteria. Multinomial logistic regression was performed to assess the correlation between anemia and aging. Cox proportional hazards models were used to evaluate the impact of anemia on all-cause mortality.

**Results:**

In 2011, anemia prevalence was 15.05% (95% CI, 14.00–16.06%) and increased with age. Age (OR, 1.03; 95% CI, 1.01–1.05; *P* < 0.01), living in rural areas (OR, 1.51; 95% CI, 1.18–1.92; *P* < 0.01), and hypoproliferation ((OR, 1.43; 95% CI, 1.12–1.83; *P* < 0.01) were positively associated with anemia.Negative associations were observed for higher hematocrit (OR, 0.93; 95% CI, 0.91–0.95; *P* < 0.01) , total cholesterol (OR, 1.00; 95% CI, 0.99–1.00; *P* = 0.03), and the creatinine clearance rate (OR, 0.99; 95% CI, 0.98–1.00; *P* < 0.01). During the 9 years of follow-up, 741 of the 4767 (15.54%) participants died. After adjusting for covariates, anemia remained independently associated with mortality (HR, 1.22, 95% CI, 1.02, 1.47; P=0.03) was still closely associated with mortality. Persistent anemia significantly impaired survival, and anemia was linked to reduced quality of life.

**Conclusion:**

This study revealed that anemia is prevalent among individuals aged ≥ 60 years in China and is related to age, residence type, hypoproliferation, hematocrit, total cholesterol and the creatinine clearance rate. Research has also confirmed that anemia is associated with worse overall survival in Chinese older adults.

**Supplementary Information:**

The online version contains supplementary material available at 10.1186/s12877-025-06353-2.

Anemia is an important global health issue. Although the clinical manifestations and complications of anemia vary on the basis of its type and severity, anemia can lead to immune dysfunction, gastrointestinal disturbances, cognitive dysfunction and impaired thermoregulation [1, 2]. Additionally, anemia may serve as a risk or prognostic factor for other diseases, such as pulmonary tuberculosis and heart failure [3, 4].

As the pace of world population growth slows and economic levels rise, the global population suffering from anemia has shown a declining trend. According to the World Health Organization (WHO) definition of anemia (hemoglobin concentrations below 130 g/L for men and 120 g/L for women) [5], the worldwide prevalence of anemia decreased from 40.2% in 1990 to 26.8% in 2019 [6, 7]. While there are certain differences in the prevalence of anemia among regions and countries due to factors such as ethnicity, economic level, and dietary habits, the overall trend is one of decline. However, the prevalence of anemia among elderly populations has been increasing annually. In 2013, the overall prevalence of anemia among Americans aged 65 and older was 17%, whereas it was only 10% in 2009 [8]. In hospitalized elderly patients, the prevalence of anemia can be as high as 50% [9]. These findings suggest that the factors contributing to anemia in older adults differ significantly from those in younger populations, necessitating tailored health guidance for elderly individuals. Many scholars believe that the increasing incidence of anemia among the elderly may be related to aging-related chronic inflammation, nutritional deficiency and the increased incidence of other diseases that may cause anemia in elderly individuals. The complex etiology of anemia in older adults, along with unique pathological mechanisms and the tendency to be comorbid with multiple diseases, severely impacts the quality of life of elderly individuals and can lead to more severe clinical outcomes. Anemia in older adults stems from multifactorial pathways including chronic inflammation, nutritional deficiencies, and comorbidities, collectively increasing mortality risk through hypoxia-induced organ dysfunction, muscle weakness, and physical frailty. Prospective studies demonstrate that persistent anemia doubles the risk of functional decline complications such as falls [10, 11] and independently elevates cardiovascular mortality by 34% [12], independent of comorbidities. Notably, it correlates with 28% higher cancer-specific mortality in treatment-resistant malignancies and synergistically increases all-cause mortality by 52% when coexisting with frailty [13, 14]. These persistent associations—despite therapeutic interventions—underscore anemia’s critical role as both a prognostic indicator and therapeutic target in geriatric care.

From a global perspective, China shares the health challenges related to anemia faced by other countries with rapidly aging populations. China stands out, however, as it already has the world’s largest older population, and the burden of anemia among the elderly in China will increase further. Research by Chinese scholars indicates that the prevalence of anemia in the urban community-dwelling elderly population in Beijing is 14.8%, increasing to 24.1% in those aged 80 years and above [15]. Additionally, the anemia diagnostic criteria used in the Chinese healthcare system (hemoglobin concentrations below 120 g/L for men and 110 g/L for women) are lower than the WHO standards, indicating that the number of elderly individuals in China requiring intervention for anemia may be greater than previously reported. Therefore, identifying the risk factors for anemia in the aging population, early identification of at-risk populations, and development of targeted screening and intervention measures are crucial. The China Health and Retirement Longitudinal Study (CHARLS) is an ongoing longitudinal survey that conducts multiple hemoglobin measurements, health function assessments, and long-term follow-ups in cohort populations, providing a valuable opportunity for research on Chinese elderly individuals [16].

This study analyzes the prevalence and trends of anemia among individuals aged 60 years and above in China on the basis of CHARLS data, explores the risk factors for anemia in elderly individuals, and identifies potential patients with anemia. Through correlational analyses of the prognosis of elderly patients with anemia, this study enhances the understanding of the impact of anemia on the survival of older adults, aiding in the management of anemia in this population and improving their quality of life and life expectancy.

## Research methods

### Study population

Our research is based on CHARLS data, a nationally representative longitudinal cohort study targeting individuals aged 45 years and above. The baseline survey (Wave 1) was conducted between 2011 and 2012, with follow-ups every 2–3 years. Data from 2011 to 2020 are publicly available. Individual and household information was collected via computer-assisted personal interviews (CAPIs). The cohort included 25,586 respondents in the baseline survey. The Biomedical Ethics Review Committee of Peking University (IRB00001052–1015) approved the design and methodology of the study, and all participants provided informed consent. For more information, please visit the CHARLS project website. We collected data on basic information, health status assessments, physical examinations, and blood tests (only in 2011 and 2015). The cohort study recruited 5,050 eligible participants for cross-sectional analysis, excluding those with a missing birth year (*N* = 297), sex (*N* = 8), hemoglobin (*N* = 3806), or younger than 60 years (*N* = 16,425). Among the 5,050 participants in the baseline survey, 1,810 were missing a Hb concentration test, and 444 were diagnosed with anemia in 2011. Therefore, our analytic sample for exploring the risk factors for anemia in elderly individuals included 2,790 participants.

### Blood sampling and biochemical analysis

CHARLS collected blood samples from all eligible participants during the baseline survey in 2011 and the follow-up survey in 2015. Trained medical personnel collected fasting blood samples at centralized facilities (district-level disease control centers in urban areas, county disease control centers, or town/village clinics in rural areas) for tests, including complete blood count (CBC), mean corpuscular volume (MCV, fL), hematocrit, and hemoglobin concentration (g/dL). Blood samples were centrifuged to separate the plasma and cellular components and then frozen at −20 °C. These samples were transported to the Chinese Center for Disease Control and Prevention in Beijing within two weeks and stored at −80 °C in ultralow-temperature freezers. Subsequent analyses were conducted on blood samples to determine the levels of various biochemical markers, including serum creatinine, glucose, lipids, and C-reactive protein (CRP). The Capital Medical University Right Yanmeng Clinical Laboratory Center completed the testing procedures. The WHO defines anemia as hemoglobin concentrations below 13 g/dL for men and below 12 g/dL for women.

### Other covariates

The relevant confounding factors, including demographic and behavioral characteristics and disease-related factors, were collected from the baseline data. The primary factors included age, sex, education level, marital status, smoking status, alcohol consumption, social activity participation, and comorbidities. Additionally, the residence variable included rural and urban areas. Body mass index (BMI) was calculated via the following standard formula: weight (kg) divided by height (m) squared (kg/m²). Inflammation was defined as a CRP concentration ≥ 10 mg/L and/or white blood cell (WBC) count ≥ 15 × 10⁹/L. Other types of cytopenia (hypoproliferation) were defined as a WBC count < 3.0 × 10⁹/L and/or a PLT < 150 × 10⁹/L. Considering the susceptibility of patients to various diseases, we collected histories of other comorbidities self-reported by patients, including hypertension, diabetes, dyslipidemia, kidney disease, heart disease, stroke, asthma, etc., totaling 14 different types of chronic diseases [16].

### Survival and health functional status

The death information and the outcome variable for each participant were sourced from death registration and certification documents, obtained through inquiries with their relatives or community managers during waves 2 through 5 of the CHARLS study conducted in 2013, 2015, 2018, and 2020. The survival time for each participant was then computed based on the outcome, with the survey year serving as the cutoff point for those whose participation was censored, thereby representing their survival duration.

### Statistical analyses

Baseline characteristics by the presence or absence of anemia are presented as the means and standard deviations for normally distributed continuous variables and percent prevalence for dichotomous variables. Baseline characteristics were compared across groups via t tests, χ^2^ tests, or Wilcoxon rank-sum tests, as appropriate. On the basis of prior CHARLS data and the correlation of covariates (*p* < 0.05) with anemia indicators, we utilized multilevel logistic regression analysis to investigate the associations among baseline age; residence type; hypoproliferation; hematocrit, total cholesterol, and creatinine clearance and anemia incidence in the population without baseline anemia. Further, this model was adjusted for baseline hemoglobin. We used Cox proportional hazards models, adjusting for age, sex, residence type, smoking status, alcohol use, hypertension status, heart disease status, diabetes status, cancer status, stroke status, cancer status, arthritis status, kidney disease status, and gastrointestinal diseases, to analyze the relationships between anemia and all-cause mortality. What’s more, We sensitivity analysis by recording participants whose survival data were missing to ensure the impact of anemia on mortality. To evaluate the impact of anemia trajectories on elderly survival, we classified participants based on hemoglobin measurements at baseline and follow-up: Persistent anemia was defined as meeting WHO anemia criteria (Hb < 12 g/dL for women; <13 g/dL for men) at both time points.Incident anemia was defined as meeting anemia criteria at at either baseline or follow-up (but not concurrently at both assessments). Also, we explored the associations between anemia and health-related quality of life indicators via χ^2^ tests. All analyses were conducted via SPSS version 27.0 (IBM Corp, Armonk, NY, USA). Statistical significance was set at *p* < 0.05, with all tests being 2 sided.

## Results

**Fig. 1:**
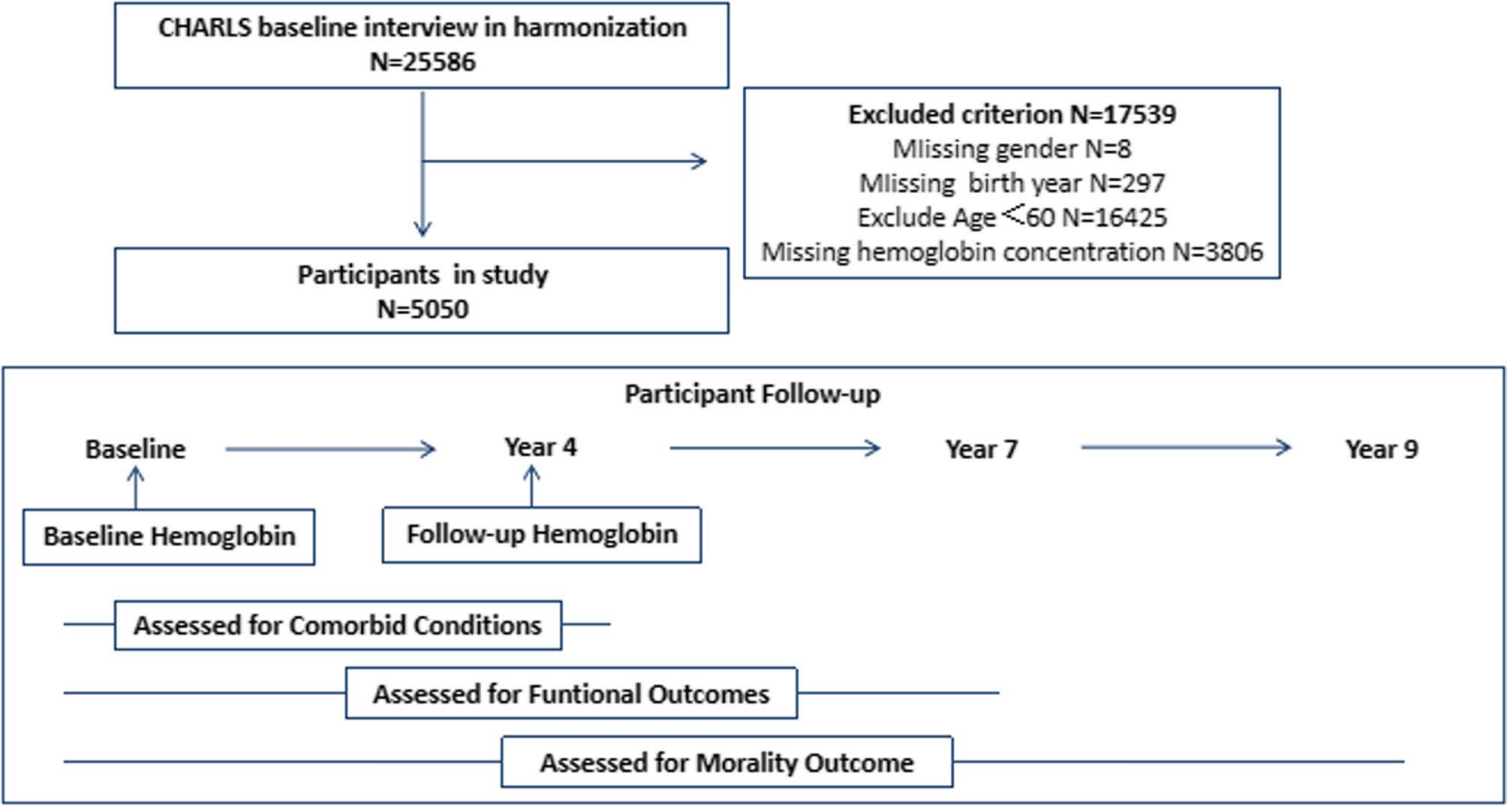
Study Schema: Participant Exclusion and Follow-up

A total of 5,050 participants met the inclusion and exclusion criteria, including 2,544 males (50.38%) and 2,506 females (49.62%). Details of the research sample selection procedure are illustrated in Fig. 1. The mean (standard deviation) age at baseline was 67.96 (6.55) years [male, 68.00(6.37); female, 67.92(6.72)], ranging from 60 to 101 years. Our findings revealed that anemia was more prevalent among older, rural residents and those with a lower BMI, income, education, inflammation, hypertension and hypoproliferation. Additionally, lower blood pressure and grip strength, slower walking speed, and reduced lung function are more common in anemia patients. In addition, the anemia group differed significantly from the nonanemia group in terms of MCV, hematocrit, total cholesterol, triglycerides, the creatinine clearance rate, uric acid, and glycated hemoglobin as well as chronic diseases, including hypertension, heart problems and stroke. The relevant baseline characteristics are shown in Table 1 for anemic and nonanemic participants from the population-based Lifeline cohort.

**Table 1:** Summary statistics of the cohort at baseline by incident anemia (WHO criteria) at baseline or four years later, resented as n (%) with χ^2^-test *p*-values or mean (standard deviation) with two-sample t-test *p*-values unless otherwise indicated

	Cohort	Incident Anemia in 2011(WHO criteria)		*P*	Cohort	Incident Anemia in 2015(WHO criteria)		*P*
	*n* = 5050	Yes *n* = 760 (15.05%)	No *n* = 4290 (84.95%)		*n* = 2796	Yes *n* = 508 (18.17%)	No *n* = 2288 (81.83%)	
Age(years)	67.96(6.55)	70.10(7.27)	67.58(6.34)	< 0.01	66.68(5.69)	68.05(6.13)	66.37(5.54)	< 0.01
Male gender	2544(50.38%)	390(51.32%)	2154(50.21%)	0.57	1404(50.21%)	258(50.79%)	1146(50.09%)	0.78
BMI	23.26(12.34)	21.79(3.28)	23.52(13.30)	< 0.01	23.87(17.17)	23.49(22.28)	23.95(15.78)	0.60
Smoker	2175(43.07%)	348(45.79%)	1857(43.29%)	0.49	1234(44.13%)	205(40.35%)	1029(44.97%)	0.06
Drink alcohol	2045(40.50%)	310(40.79%)	1735(40.44%)	0.81	1166(41.70%)	185(36.42%)	981(42.88%)	0.01
Less than lower secondary	4786(94.77%)	733(96.45%)	4053(94.48%)	0.07	2667(95.39%)	495(97.44%)	2172(94.93%)	0.04
Living in rural village	3315(65.64%)	520(68.42%)	2795(65.15%)	0.08	1898(67.88%)	383(75.39%)	1515(66.22%)	< 0.01
Pension income(thousand)	4.09(10.09)	2.98(8.03)	4.28(10.40)	< 0.01	3.72(9.86)	2.53(7.63)	3.98(10.27)	< 0.01
BP(systolic/diastolic)	134.29(22.57)/74.58(11.78)	132.10(22.81)/71.56(11.94)	134.69(22.51)/75.12(11.67)	< 0.01	74.82(11.51)/133.80(22.17)	72.60(11.89)/130.50(21.89)	75.32(11.37)/134.54(22.17)	< 0.01
pulse	72.05(10.66)	71.30(10.30)	72.18(10.72)	0.05	71.91(10.56)	71.69(10.68)	71.96(10.53)	0.09
Time for walking speed test(sec)	4.67(2.45)	4.94(2.42)	4.62(2.45)	< 0.01	4.48(2.25)	4.82(2.38)	4.40(2.21)	< 0.01
Grip strength(kg)	28.37(10.19)	26.92(10.00)	28.64(10.20)	< 0.01	29.34(9.99)	27.62(8.94)	29.72(10.17)	< 0.01
Lung function peak flow	253.66(116.95)	242.18(110.98)	255.75(117.90)	< 0.01	261.46(116.71)	248.48(110.69)	264.38(117.85)	0.01
Hypertension	1727(34.20%)	207(27.24%)	1520(35.43%)	< 0.01	970(34.69%)	162(31.89%)	808(35.31%)	0.15
Diabetes mellitus	368(7.29%)	50(6.58%)	318(7.41%)	0.42	208(7.44%)	40(7.87%)	168(7.34%)	0.67
Cancer	40(0.79%)	9(1.18%)	31(0.72%)	0.18	21(0.75%)	5(0.98%)	16(0.70%)	0.50
Heart problem	787(15.58%)	94(12.37%)	693(16.15%)	0.01	438(15.67%)	62(12.20%)	376(16.43%)	0.02
Stroke	204(4.04%)	40(5.26%)	164(3.82%)	0.06	91(3.25%)	20(3.94%)	71(3.10%)	0.34
Arthritis	1905(37.72%)	281(36.97%)	1905(44.41%)	0.78	1049(37.52%)	208(40.94%)	841(36.76%)	0.08
Kidney disease	322(6.38%)	44(5.79%)	278(6.48%)	0.49	175(6.26%)	39(7.68%)	136(5.94%)	0.15
Stomach/digestive disease	1099(21.76%)	163(21.45%)	936(21.82%)	0.85	623(22.28%)	120(23.62%)	503(21.98%)	0.43
Inflammation(CRP ≥ 10 or WBC ≥ 15)	298(5.90%)	65(8.55%)	233(5.43%)	< 0.01	133(4.76%)	28(5.51%)	105(4.59%)	0.34
Hypoproliferation(WBC < 3.0 or PLT < 150)	1134(22.46%)	214(28.16%)	920(21.45%)	< 0.01	580(20.74%)	138(27.17%)	370(16.17%)	< 0.01
Hemoglobin (g/dl)	14.27(2.20)	11.39(1.20)	14.78(1.93)	< 0.01	14.81(1.96)	14.08(1.57)	14.97(2.00)	< 0.01
White blood cell in thousands	6.26(1.95)	5.88(2.10)	6.32(1.91)	< 0.01	6.32(1.91)	6.36(2.13)	6.31(1.86)	0.54
Platelets(×10^3^/µL	206.79(75.67)	206.77(90.64)	206.80(72.71)	0.99	208.77(73.85)	199.07(72.15)	210.92(74.06)	< 0.01
MCV	91.36(8.57)	87.48(11.53)	92.05(7.73)	< 0.01	91.92(7.52)	91.86(8.69)	91.94(7.25)	0.82
Glucose (mg/dl)	112.38(38.83)	110.71(38.80)	112.68(38.84)	0.20	112.33(35.84)	111.35(37.86)	112.54(35.39)	0.50
Total cholesterol (mg/dl)	194.35(38.78)	180.40(38.56)	196.85(38.56)	< 0.01	197.04(38.45)	192.73(36.40)	197.99(38.84)	< 0.01
Triglycerides (mg/dl)	128.47(99.33)	109.50(81.80)	131.87(101.79)	< 0.01	130.45(102.49)	122.72(113.06)	132.15(99.95)	0.06
Glycated hemoglobin (%)	5.31(0.83)	5.20(0.80)	5.33(0.84)	< 0.01	5.32(0.84)	5.31(0.85	5.33(0.84)	0.65
Uric Acid (mg/dl)	460(1.31)	4.69(1.44)	4.58(1.29)	0.06	4.53(1.23)	4.54(1.21)	4.52(1.23)	0.76
Hematocrit	41.43(6.11)	34.85(5.07)	42.60(5.50)	< 0.01	42.67(5.67)	40.85(5.69)	43.07(5.59)	< 0.01
Creatinine clearance rate(%)	67.42(19.69)	61.27(19.80)	68.51(19.47)	< 0.01	70.62(19.35)	65.492(18.01)	71.66(19.48)	< 0.01

**Fig. 2:**
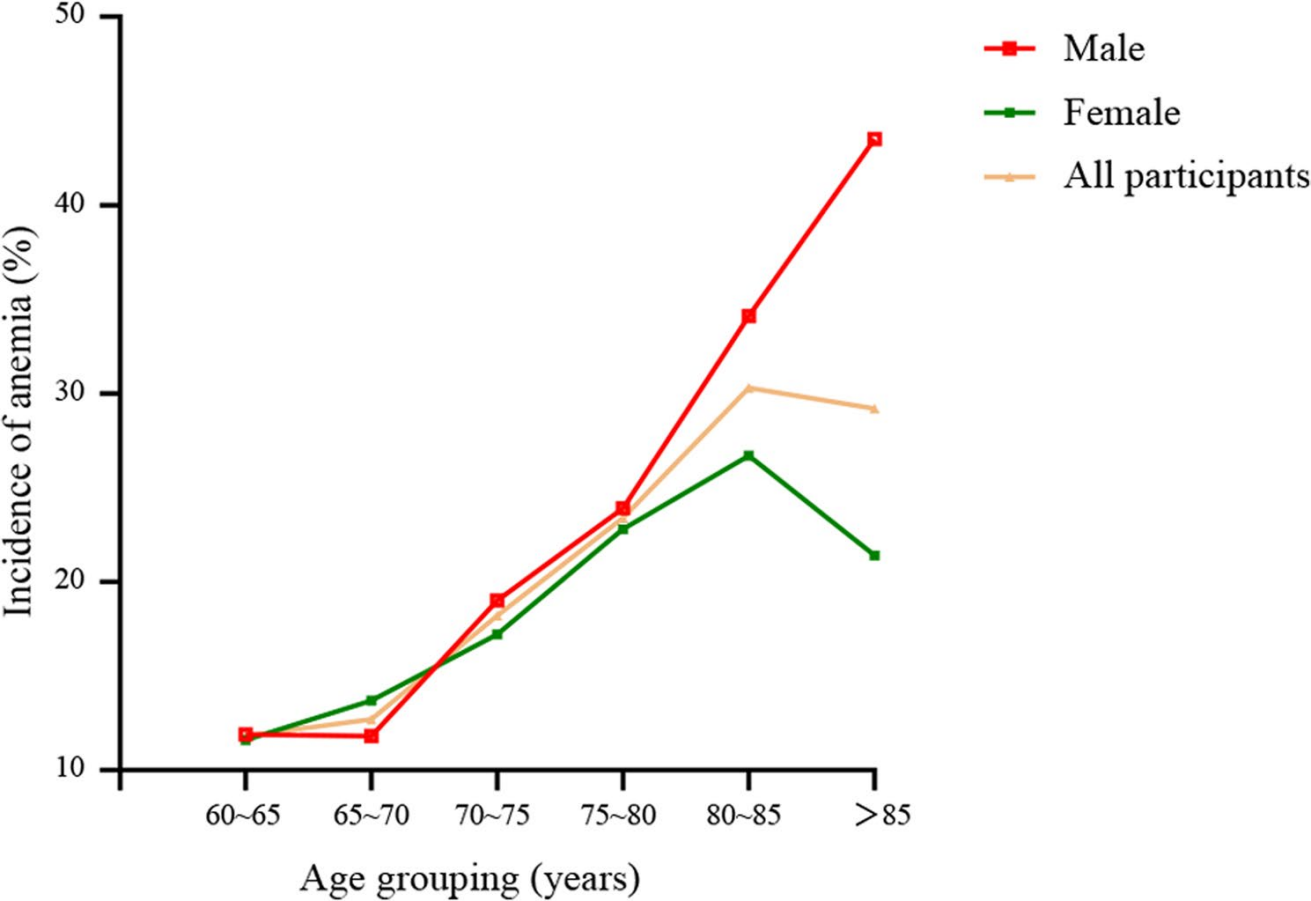
The prevalence of anemia as a function of sex and age

At baseline, a total of 390 men and 370 women had anemia according to the WHO definition, whereas the overall prevalence of anemia in this population was 15.05%(95% CI, 14.00%, 16.06%). Among women, the overall prevalence of anemia was 30.34%, with the highest prevalence (26.67%) observed in the 80–85 years age cohort. In contrast, the prevalence of anemia among males increased gradually with age, peaking at 43.48% in those aged over 85 years (Table 2; Fig. 2). Among the 3240 participants who had follow-up hemoglobin results, 761 had anemia. The mean (standard deviation) baseline hemoglobin level was 14.27 (2.20) g/dL: 14.84 (2.18) g/dL for men and 13.69 (2.08) g/dL for women. The median 4-year change (interquartile range) in hemoglobin was − 0.80 (−1.80, 0.30) g/dL; for males, it was − 0.80 (−1.90, 0.40) g/dL; and for females, it was − 0.80 (−1.80, 0.20) g/dL. Among individuals who developed anemia, 64.91% experienced a decrease in hemoglobin greater than 1 g/dL. However, only 34.62% of those with a decrease in hemoglobin greater than 1 g/dL developed anemia during the follow-up period.

**Table 2:** Prevalence of anemia in individuals older than 60 years

	Incident Anemia				Anemia prevalence		
	Yes *n* = 760		No *n* = 4290				
Age	Male	Female	Male	Female	Male	Female	Cohort
60 ~ 65	134	132	990	1005	11.92%	11.61%	11.76%
~ 70	73	84	548	531	11.76%	13.66%	12.70%
~ 75	83	64	354	308	18.99%	17.20%	18.17%
~ 80	60	57	191	193	23.90%	22.80%	23.35%
~ 85	30	24	58	66	34.09%	26.67%	30.34%
> 85	10	9	13	33	43.48%	21.43%	29.23%

Among the 3,240 participants with follow-up hemoglobin results, 2,796 did not meet the WHO definition of anemia at baseline. In this population, the morbidity of anemia was 18.17% (*N* = 508). Using the presence or absence of anemia at follow-up as the dependent variable, we performed a multilevel logistic regression analysis on this population without anemia at baseline (*N* = 2796) to identify risk factors for anemia in older adults. The multivariable logistic regression model incorporated several factors: age, residence type, education level, alcohol consumption, heart problems, hypoproliferation, hematocrit, total cholesterol and creatinine clearance. Notably, age (OR, 1.03; 95% CI, 1.01–1.05; *P* < 0.01), living in rural areas (OR, 1.51; 95% CI, 1.18–1.92; *P* < 0.01), hypoproliferation (OR, 1.43; 95% CI, 1.12–1.83; *P* < 0.01), hematocrit (OR, 0.93; 95% CI, 0.91–0.95; *P* < 0.01), low total cholesterol (OR, 1.00; 95% CI, 0.99–1.00; *P* = 0.03) and creatinine clearance (OR, 0.99; 95% CI, 0.98–1.00; *P* < 0.01) were associated with anemia incidence (Table 3). After adjustment for baseline hemoglobin, the relationships between these factors and anemia incidence remained unchanged. However, no significant associations were detected for sex (*P* = 0.78), body mass index (*P* = 0.60), hypertension (*P* = 0.15), or stomach/digestive disease (*P* = 0.43). Table 4.

**Table 3:** OR or AOR of incident anemia associated with age, residence type, hypoproliferation, hematocrit, total cholesterol and creatinine clearance rate

	Model 1		Model 2^a^	
	OR(95%CI)	*P*	AOR(95%CI)	*P*
Age	1.03(1.01, 1.05)	< 0.01	1.03(1.01, 1.05)	< 0.01
Residence type	1.51(1.18, 1.92)	< 0.01	1.61(1.26, 2.06)	< 0.01
Hypoproliferation	1.43(1.12, 1.83)	< 0.01	1.62(1.26, 2.09)	< 0.01
Hematocrit	0.93(0.91, 0.95)	< 0.01	0.97(0.94, 1.00)	0.01
Total Cholesterol	1.00(0.99, 1.00)	0.03	1.00(0.99, 1.00)	0.03
Creatinine clearance rate	0.99(0.98, 1.00)	< 0.01	0.99(0.98, 1.00)	< 0.01

^a^ Adjusted for baseline hemoglobin

OR, odds ratio; AOR, adjusted odds ratio; CI, confidence interval

**Table 4:** Multivariate-adjusted hazard ratio (HR) for mortality due to anemia

	HR (95%CI) in Anemia	*P*
	4767	
No. of deaths	741	
Model A	1.59(1.34,1.90)	< 0.01
Model B	1.24(1.03,1.48)	0.02
Model C	1.22(1.02,1.47)	0.03

Model A: anemia

Model B: Model 1 + age, sex, residence type, smoking status, alcohol consumption status

Model C: Model 3 + hypertension, heart problems, diabetes mellitus, cancer, stroke, arthritis, kidney disease, and stomach/digestive disease

**Fig. 3:**
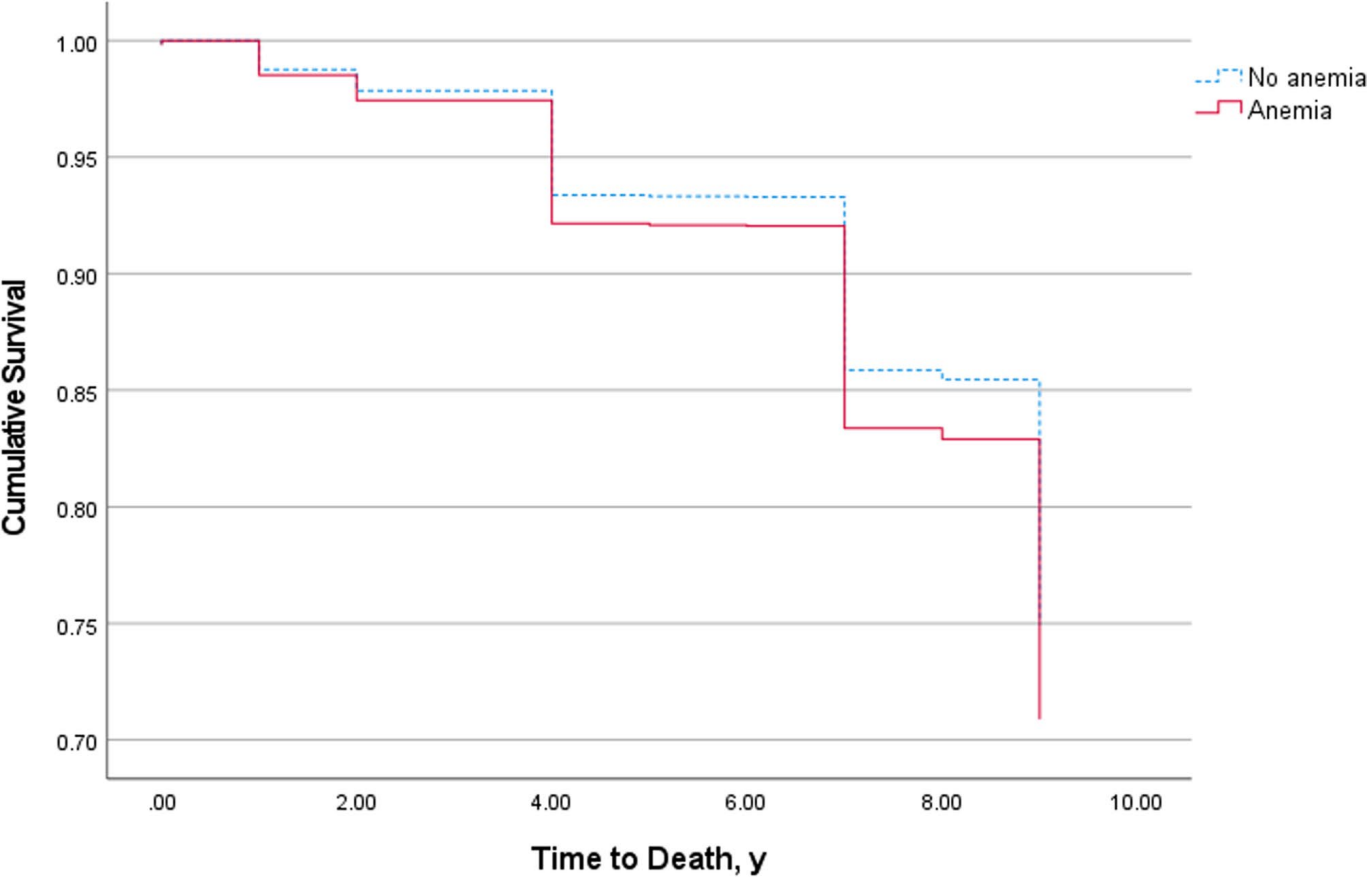
Overall survival in individuals older than 60 years

During the 9-year follow-up Figure 3. (data from wave 2–5), a total of 283 of 5,050 (5.60%) people failed to attend the visit. Thus, a cohort of 4767 individuals was included in the longitudinal cohort analysis. 741 (15.54%) of the 4767 participants died. After adjustment for age, sex, residence type, smoking status, alcohol consumption, hypertension, heart problems, diabetes, cancer, stroke, arthritis, kidney disease and stomach/digestive disease, incident anemia (HR, 1.22, 95% CI, 1.02, 1.47; *P* = 0.03) was associated with increased mortality. Moreover, elderly individuals with anemia have poorer functional status and quality of life (Table 5). Multivariate logistic regression analysis demonstrated that anemia serves as an independent risk factor for difficulty with dressing (*P* = 0.03) and getting into or up of bed (*P* = 0.03) the Chinese elderly population (Table 6).

**Table 5:** The occurrence of physical degeneration and dificullty in daily life during follow-up period by incident anemia (WHO criteria) at baseline, resented as n (%) with χ^2^-test *p*-values

		Incident Anemia in 2011(WHO criteria)		*P*
	Cohort *n* = 5050	Yes *n* = 760	No *n* = 4290	
Hip fracture	181(3.58%)	34(4.47%)	147(3.43%)	0.04
Teeth loss	309(6.12%)	54(7.11%)	258(6.01%)	0.02
Difficulty with dressing	510(10.10%)	86(11.32%)	424(9.88%)	0.01
Difficulty with getting into or out of bed	522(10.34%)	92(12.11%)	430(10.02%)	< 0.01
Difficulty with controlling urination/Defecation	344(6.81%)	61(8.03%)	283(6.60%)	0.01
Difficulty with shopping for groceries	720(14.26%)	115(15.13%)	605(14.10%)	0.03
Difficulty with making phone calls	1369(27.11%)	213(28.03%)	1156(26.98%)	0.01

**Table 6:** Logistic regression models for anemia and functional limitations ^a^ adjusted for age, sex, residence type, smoking status, alcohol consumption status, hypertension, heart problems, diabetes mellitus, cancer, stroke, arthritis, kidney disease, and stomach/digestive disease

	Model 1		Model 2^a^	
	OR(95%CI)	*P*	AOR(95%CI)	*P*
Difficulty with dressing	1.39(1.08, 1.80)	0.01	1.34(1.02,1.76)	0.03
Difficulty with getting into or out of bed	1.49(1.16, 1.92)	< 0.01	1.36(1.04, 1.78)	0.03

^a^ Adjusted for age, sex, residence type, smoking status, alcohol consumption status, hypertension, heart problems, diabetes mellitus, cancer, stroke, arthritis, kidney disease, and stomach/digestive disease.

**Fig. 4:**
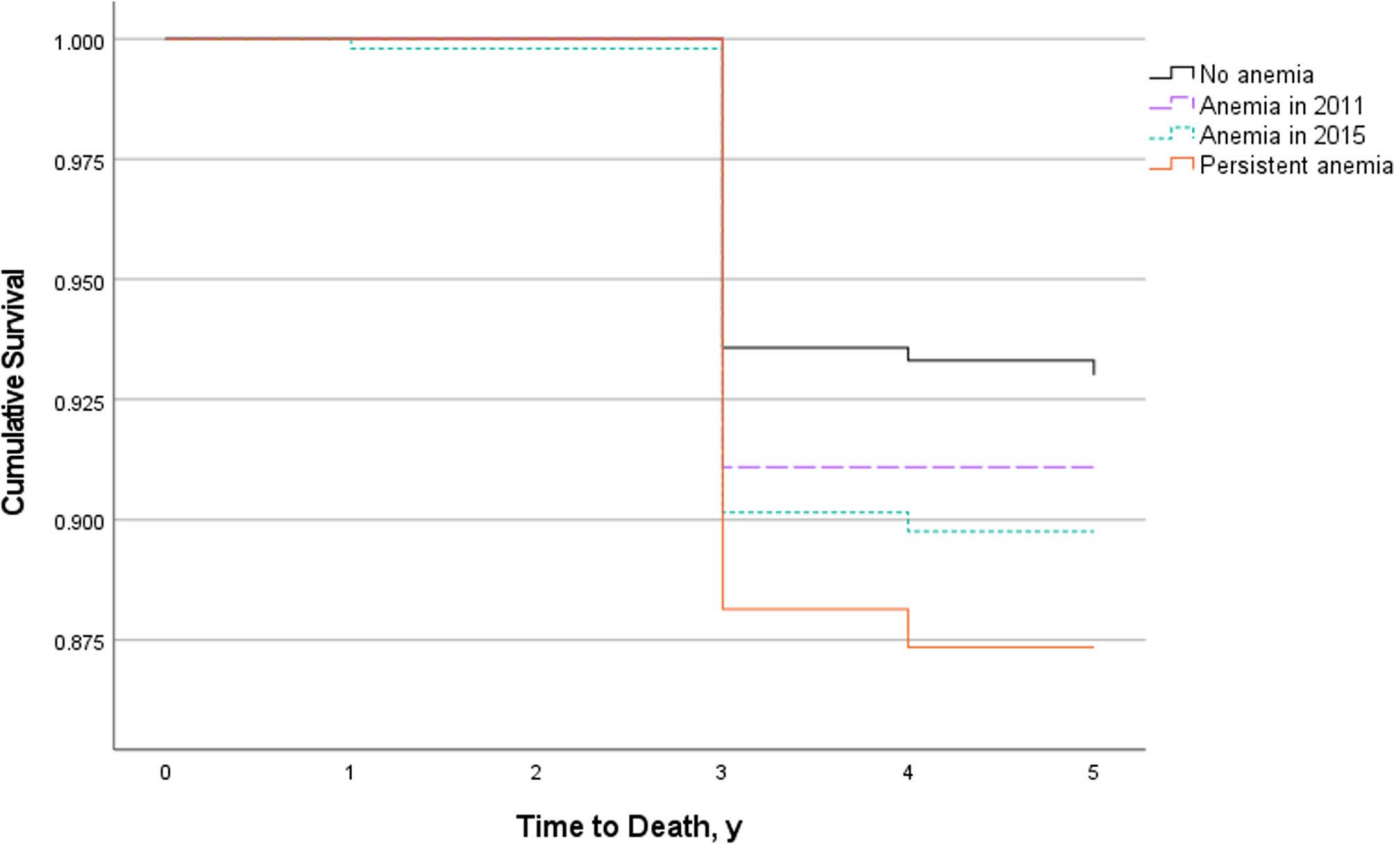
Overall survival in individuals with no anemia, incident anemia or persistent anemia

As shown in Table 7, among the 3,240 patients whose follow-up hemoglobin data were available, 444 participants (13.70%) had anemia at their index visit. Among those who had anemia at their index visit, 253 participants (56.98%) still met the anemia criteria at follow-up. In accordance with the definition outlined in the Methods section, these participants were classified as having persistent anemia. We found that the duration of anemia also impacted the survival of the elderly population (data from wave 3–5), with persistent anemia group (HR, 1.51; 95% CI, 1.02–2.22; *P* = 0.04) having a greater impact compared to other groups (Fig. 4, Supplementary 2).

**Table 7:** Anemia status at the index and follow-up visits

	Follow-Up visit		
Index Visit	Anemia Status	Yes	No
	Yes	253	191
	No	508	2288

## Discussion

In this community-based study of 5,050 older adults, anemia was common and increased with age. A significant association was found between anemia and age, residence type, hypoproliferation, hematocrit, total cholesterol and creatinine clearance.There is a significant correlation between elderly anemia and all-cause mortality. Further, the impact of anemia on mortality was worse with a longer duration of anemia. Among elderly individuals with anemiaare more prone to functional limitations.

The prevalence of anemia among older adults in this study was 15.05% in 2011 and 22.02% (data not shown) in 2015, exceeding the previously reported national figure of 12.86% [17]. A plausible explanation for this discrepancy could involve a participation bias within the Lifelines cohort, despite prior observations indicating that the Lifelines study population is representative of the general population, with a baseline mean age of 59.13 (9.66) years. Similar to other studies, the prevalence of anemia among elderly individuals in China is increasing. Furthermore, the prevalence of anemia among older adults, particularly males, increases with age. Compared with that in the 60–65 age group, the prevalence of anemia in those over 85 years of age was approximately twice as high (29.23% vs. 11.76%), indicating a need to increase the frequency of anemia screening in this high-risk group. In men aged ≥ 85, anemia significantly elevates mortality risk through synergistic mechanisms: chronic inflammation suppresses erythropoiesis and iron metabolism, while age-related testosterone deficiency exacerbates erythropoietin resistance and sarcopenia, impairing oxygen delivery and physical resilience. These findings align with studies in octogenarians, including Carrascal Y et al. [18], which reported a 2.78-fold increase in postoperative mortality among anemic men aged ≥ 85, and Zheng Z et al. [19], who linked low testosterone and anemia to higher mortality risk in elderly men. Notably, anemia in the oldest-old (≥ 85 years) often stems from irreversible drivers such as chronic kidney disease and clonal hematopoiesis, unlike reversible causes in younger elderly populations. This etiological divergence underscores the need for age-specific diagnostic strategies and targeted therapies to reduce mortality risks in this vulnerable cohort.

Between the years 2011 and 2015, the incidence of anemia among the cohort was 18.07%. Despite observing distinct trends in anemia prevalence among elderly individuals of varying genders, our analysis did not reveal a significant association between gender and anemia in this population. Nevertheless, we did identify a notable difference in anemia rates across different residence types, with a higher proportion of anemia cases observed among elderly individuals residing in rural areas. China still classifies as a developing nation, with its residents’ urbanization process still ongoing and not yet finalized. There are significant disparities between rural and urban elderly residents in terms of population quality, living standards, and healthcare insurance. These factors could facilitate the development of anemia [20], And identify it as a contributing risk factor for the incidence of anemia among the Chinese elderly population.

The process of aging typically coincides with the deterioration of organ functions and the onset of systemic, age-related inflammation. Within this scenario, the bone marrow proliferative capacity diminishes in elderly individuals, resulting in the accumulation of senescent cells. Consequently, this establishes an adverse milieu characterized by excessive HSC proliferation, loss of pluripotency, and immune system depletion, thereby elevating the risk of anemia [21–27]. Oxidative imbalance that arises during the aging process can further induce abnormalities in cholesterol metabolism, damaging the structure of red blood cells. This, in turn, results in erythropoiesis disorders and hemolysis, thereby elevating the risk of anemia among the elderly population [28–32]. Purnamasari SD et al. [33] and Ni W et al. [34] have confirmed the correlation between dyslipidemia and anemia incidence. Although our results indicate a potential association between total cholesterol and anemia in the elderly, the narrowness of the confidence interval, which is very close to 1, necessitates further research to solidify this correlation.

At least one-third of anemic patients aged 65 and older exhibit a hyperinflammatory state characteristic of chronic kidney disease (CKD). Reduced erythropoietin (EPO) production, insufficient to counteract anemia, and a diminished response of erythroid progenitors to EPO are crucial underlying mechanisms. Haslam et al. [35] identified decreased renal function (eGFR < 45 mL/min/1.73 m^2^) as a significant predictor of anemia in centenarians. Our study, along with others [12, 36], corroborates this finding. Conversely, anemia can exacerbate kidney function decline, primarily through hypoxia and/or heightened oxidative stress [37]. Thus, a vicious cycle emerges, where worsening anemia perpetuates worsening CKD, posing a significant health threat to the elderly population.

The aging population and the increasing prevalence of anemia among older adults will greatly increase the number of anemic elderly individuals in China. Therefore, understanding the relationships between anemia and quality of life and/or longevity is crucial. Thein et al. [38] reported that anemia in individuals aged 65 and older was independently associated with impairments across multiple subscales of health-related quality of life in a cohort of 328 subjects in the United States. Doni L et al. [39] reported a correlation between quality of life, length of hospitalization, and anemia in older cancer patients. These were small studies focused on older adult populations in Europe and America. Our study confirms that anemia impacts daily quality of life and mortality among older adults in China, with persistent anemia having a more severe effect on all-cause mortality. Remarkably, there has been limited research examining changes in anemia status over time among older adults. In our study, we noted a concerning trend: persistent anemia was associated with increased mortality.

There are three major potential implications of this study. Firstly, our study, with a large sample size and rigorous quality control measures, provides valuable insights into the prevalence of anemia and identifies risk factors for anemia among the Chinese elderly, ensuring data validity and reliability. Secondly, our findings underscore the importance of advocating for early hemoglobin testing in the elderly, given the association between anemia and increasing age, to facilitate prevention or early diagnosis. Additionally, renal function improvement programs should be incorporated into guidelines for managing anemia in the elderly, as poor kidney function poses a significant health risk, regardless of anemia status, as evidenced by this and other studies. Thirdly, anemia in the elderly should be considered a marker for subsequent adverse outcomes. To explore the intricate relationship between anemia and confounding factors, our study analyzed the potential association between anemia and mortality, adjusting for factors such as age, behavioral characteristics, and comorbidities. In summary, anemia is a relevant prognostic indicator. Therefore, anemia in older individuals should be taken seriously and thoroughly investigated.

The limitations of our study include that CHARLS is an observational cohort. Additionally, this database does not contain information on anemia treatment, making it impossible to explore the associations between anemia treatment and adverse outcomes in older adults with anemia. Second, we only have hemoglobin levels from two time points four years apart, and more frequent hemoglobin testing is needed to accurately describe the effects of anemia status on the quality of life and mortality of older adults. Third, while this cohort study is meticulously designed to assess the relationship between potential risk factors and anemia in the elderly, as well as the relationship between anemia in the elderly and health outcomes, our research hinges on data pertaining to the general characteristics, behaviors, and disease-related aspects of the sample population. These data were collected using a questionnaire developed by CHARLS and may contain inaccuracies due to factors such as faulty recall, the elapsed time since events, and potential biases stemming from current cognitive status. Finally, while our analysis emphasizes chronic inflammation, renal dysfunction, and residential care as mediators of anemia-related mortality, we acknowledge gaps in addressing other critical contributors. Notably, malnutrition, cancer-associated anemia, and hematologic malignancies—conditions prevalent yet understudied in community-based cohorts of the oldest-old—likely exacerbate mortality risk through mechanisms such as bone marrow suppression, clonal hematopoiesis, or cytokine-driven erythropoietin resistance. Consequently, these inaccuracies could potentially undermine the precision of our study’s findings.

In summary, anemia is prevalent among older adults in China. Age, residing in rural areas, hypoproliferation, hematocrit, total cholesterol levels and a decline in creatinine clearance are correlated with anemia among the Chinese elderly. Notably, anemia, particularly persistent anemia, is associated with an elevated risk of mortality in Chinese elderly patients with anemia. Future research is needed to further explore the impact of hemoglobin levels and changes in hemoglobin levels on the quality of life and mortality rates of the elderly population, as well as whether interventions aimed at preventing anemia and hemoglobin changes can significantly improve mortality outcomes in this group.

## Supplementary Information

Supplementary Material 1.

## Data Availability

The data are available upon reasonable request at https://charls.charl.sdata.com/index/zh-cn.html.

## Abbreviations

CHARLS: China Health and Retirement Longitudinal Study
WHO: World Health Organization
CAPI: computer-assisted personal interviews
BP: Blood pressure measure
CBC: complete blood count
MCV: mean corpuscular volume
CRP: C-reactive protein
BMI: body mass index
WBC: white blood cell count
HSC: hematopoietic stem cell
HMG-CoA: 3-hydroxy-3-methylglutaryl-coenzyme A
EPO: erythropoietin
OR: odds ratio
AOR: adjusted odds ratio
HR: hazard ratio
AHR: adjusted hazard ratio
CI: confidence interval
CKD: chronic kidney disease
