# Supplementary material for: The prevalence and etiology of anemia and the association between anemia and all-cause mortality: a cohort study over a 9-year period
Source: BMC Geriatr. 2025 Sep 26;25:707. doi: 10.1186/s12877-025-06353-2 (PMC12465429; doi:10.1186/s12877-025-06353-2)
Supplement: Supplementary file 1 — Supplementary Material 1. [file 12877_2025_6353_MOESM1_ESM.docx]

|  | HR (95%CI) in Anemia | P |
| --- | --- | --- |
|  | 5050 |  |
| No. of deaths | 741 |  |
| Model 1 | 1.62(1.36,1.93) | <0.01 |
| Model 2 | 1.27(1.06,1.52) | 0.01 |
| Model 3 | 1.26(1.05,1.51) | 0.01 |

**Supplementary 1** Sensitivity analyses for the relationship between anemia and mortality

Model 1: anemia

Model 2: Model 1 + age, gender, residence type, smoke, drink alcohol

Model 3: Model 3 + Hypertension, Heart problem, Diabetes Mellitus, Cancer, Stroke, Arthritis, Kidney disease, Stomach/digestive disease

| Duration of anemia | N(%) | HR (95%CI) | P | AHR^a^( 95%CI) | P |
| --- | --- | --- | --- | --- | --- |
| No anemia | 2288(70.62%) | Reference | - | Reference | - |
| Anemia in 2011 | 191(5.90%) | 1.28(0.77,2.10) | 0.34 | 1.19(0.72,1.96) | 0.50 |
| Anemia in 2015 | 508(15.68%) | 1.47(1.08,2.01) | 0.02 | 1.23(0.89,1.70) | 0.21 |
| Persistent anemia | 253(7.81%) | 1.82(1.24,2.65) | <0.01 | 1.51(1.02,2.22) | 0.04 |

**Supplementary 2** HR or AHR for mortality due to duration of anemia

^a^ Adjusted for age, sex, residence type, smoking status, alcohol consumption status, hypertension, heart problems, diabetes mellitus, cancer, stroke, arthritis, kidney disease, and stomach/digestive disease

HR, hazard ratio; AHR, adjusted hazard ratio; CI, confidence interval
